# Pleurisy

**Published:** 1894-01-06

**Authors:** 


### MEDICAL PROGRESS.

### PLEURISY.

Much has been done of late to determine the cause of pleural effusions, particularly with respect to the importance of bacterial antecedents, but far more than the mere presence of bacteria in the fluid must be proved before we can regard these agents as necessary causes of effusion. There is a tendency on the one hand to regard all pleurisies as tubercular, and on the other some authorities trace many forms to non-bacterial agencies. Jakowski[1] records the results of his search for bacteria in 52 cases, and compares these with the examinations made on 248 patients by Levy, Fränkel (A.), Wetter, and others. He insists on bacteria being actually the means by which all pleurisies are caused, given the presence of favourable conditions, and that where no bacteria can be found in serous or moist purulent effusions there is a tubercular origin, but that many empyæmata are due to other forms of sepsis, especially to the streptococcus pyogenes. This latter form is marked by a greater tendency to suppuration and runs a more unfavourable course. In this, too, the pus must be removed as early and perfectly as possible, and resection of the ribs is frequently needed. A third type, which includes most of the primary idiopathic non-tubercular pleurisies—the so-called rheumatic ones—are due to Fränkel's diplococci, which soon lose their virulence, while the course of the disease is correspondingly favourable. Mixed bacteria occur in some cases, and the absence of any at all may be due to their death in the early stages of the attack.

Dr. Osler[2] in his Shattuck lecture on "Tuberculous Pleurisy," agrees that the physical characters of most tubercular effusions give no evidence of their nature, even inoculations are most frequently sterile. Thirty-two per cent. of pleurisies, where an autopsy was held, turned out to be tubercular. He would regard as suggestive of tubercle "a thin sero-purulent exudate, a little opalescent, often with a greenish tint and which microscopically contains a granular fatty matter and only a few leucocytes." Other evidence must be sought in the lungs, the glands, and in the family history, and by frequent examination of the sputa. A fluid which constantly recurs after tapping is most probably tubercular in origin. The treatment is the same for the tubercular and other varieties. The apparent absence of micro-organisms in many "cold" pleurisies is discussed by Sacaze[3] (J.), who points out that even the inoculation test may fail if too small quantities of fluid are used. He has demonstrated the presence of streptococci and tubercle in the same serous effusion, both of which disappeared, at a second aspiration a few days later, when the fluid did not recur.

In contrast with these teachings it is instructive to consider how far mechanical causes are able to produce or increase an effusion. Whether or no the beginning of exudation may not be traced to mechanical causes in some instances, its increase and progress may depend on the movements and position of the patient. Volland[4] points out that when a patient having a little fluid in his pleura lifts himself up the fluid shifts its place and exerts a gradual pressure on a fresh part of the lung, a negative pressure is produced on the side it has left, and, since expansion does not follow at once, more fluid is forced out to fill the vacuum. Hence he forbids the slightest movement to patients with small effusions, neither allowing them to turn over, nor even to sit up for purposes of examination. Even strong respiratory movements may increase the amount of fluid. He, therefore, watches the heart and respiration for any symptoms of the increase of fluid until the temperature has been normal for eight days, the patient being kept flat on his back the whole while. If any fluid remains when he is examined longer rest is ordered. Very great success follows the use of this plan in small effusions. With regard to the theory of pressures on which this argument is founded, while Douglas Powell and others have laid down that "a negative pressure is maintained in the pleura until the lung has completely contracted before the advancing fluid," T. Calvert[5] has shown by a series of experiments that fluid occupying a quarter of the pleural cavity will give a positive intra-pleural pressure, and that the lung contracts as a whole, and not by the collapse of the lowest vesicles. Homolle arrived at much the same view by the use of a manometer.

Dr. G. A. Sutherland,[6] in a valuable lecture, points out the difference between the first and second stages of effusion, that in the first the pressure of the fluid is cancelled and it is held in suspense by the elasticity of the lung which may also draw the thoracic organs out of their position and produces the S-shaped curve along the base of the contracted lung. Thus the elasticity of the lung and not a pressure of the fluid is the active agent until the second stage of extreme effusion. This is marked by depression of the liver, or dulness of Traube's space, gush of fluid through a trocar, faintness, weak pulse, &c., and implies positive intrapleural pressure.

The practical value of the manometer as an adjunct to the aspirator is insisted on by Professor Gennaro Petteruti,[7] of Naples, because the clinical signs which are usually relied upon to show when the right amount of fluid has been drawn off are not accurate, and a dangerous degree of negative pressure may be set up. He uses a slowly acting manometer, and directs that the greatest negative pressure reached during evacuation should not exceed 20 millimetres.

The question of early tapping has been discussed by Dr. Herman C. Gardiner,[8] who claims to have had more rapid and perfect results by it than under any other plan. In all moderately large effusions he aspirates even before the subsidence of the ordinary subfebrile state, which usually disappears after the operation. Of twenty cases thus treated, fourteen required only one aspiration, and in all the evacuation was useful.

Bernard Pitts,[9] in his lectures at the Royal College of Surgeons, held that "the best results are to be obtained in the treatment of empyæma in children by early free incision and effective drainage." Aspiration has special dangers, but may be adopted when the effusion is sero-purulent or very localised. In children, the removal of a portion of rib has no disadvantages and many advantages, the removal of masses of solid lymph leading to a much more rapid cure. When the entire cavity is involved an opening just below and anterior to the angle of the scapula is best, but this must depend on the special conditions of the case. In most instances irrigation is not advisable, and meddlesome interference with slowly contracting cavities is to be deprecated. In 83 cases treated by incision at Great Ormond Street and St. Thomas's Hospital there were 51 recoveries, 16 relieved, and 16 deaths; and in 129, where a rib was resected there were 99 recoveries, seven relieved, and 23 deaths. In double empyæma, we should operate as early as possible, but it is usually best to leave the second side for a few days after the first has been opened.

Watrowbu[10] warmly advocates permanent aspiration and drainage as far preferable to any other plan for serous and most purulent effusions. An incision is made through the skin. A trocar and cannula, the size of a man's little finger, having been thrust into the cavity, a thick-walled rubber tube is passed through the canula and the latter is then withdrawn. The tube is fixed in its position by a safety-pin and strapping. Some three feet of drainage tubing is attached, and the lower end carried into a disinfecting solution under the bed. After a few days, when the fever has subsided, the patient gets up and walks about, the tube being carried fitted into a bottle suspended at his side or in a pocket, its upper end being fixed as before, until the secretion has practically ceased. Minute directions as to details are given by the author, who has brought the method to great perfection.

The difficulties attending the diagnosis of diaphragmatic pleurisy is the subject of a valuable paper by Dr. Soltau Fenwick[11], where the history of five cases is given at length. He points out that "the sudden occurrence of severe pain in the lower part of the chest or hypochondriac region, increased by respiration, or by pressure with the hand, a shallow costal type of respiration with lack of movement on the part of the diaphragm, severe general symptoms associated with a total absence of physical signs, all suggest an inflammatory affection of the inferior surface of the pleura."

Thus, in a child, convulsions and opisthotonos; in adults, severe abdominal pain, with vomiting, marked the commencement of an attack. The pain is always very severe, and may be felt in the hypochondrium, iliac fossa, or elsewhere in the abdomen. There is intense distress, and often a fear of impending death. The respiratory movements are hurried, and confined to the upper part of the thorax, the abdomen very tender, and the superficial muscles firmly contracted, even the lower ribs being practically motionless; but there is generally less collapse than in ordinary peritonitis. The pain, too, is "more superficial in character than that usually encountered in cases of true visceral disease, and it is markedly increased by forced respiratory efforts," while neither tumour, increased resistance, nor rectal signs can be found. The temperature is higher than in ordinary pleurisy, but cough is not so often met with. Herpes may occur round the dry, cracked lips, and the vomiting may be serious in character. The diagnosis is often very difficult, but may be cleared up by the appearance of a friction sound at the base of one pleura followed by dulness and the signs of effusion. Then the abdominal symptoms disappear, and the general condition rapidly improves in many cases. We may have to consider the possibility of such diverse affections as biliary colic, appendicitis, and pneumonia.

Vanclair[12] reports a case of subphrenic abscess containing air in a boy aged six, complicated with empyæma, perforation of the diaphragm, and colon. Recovery followed an operation where the intestine was sutured, and the abscess and the empyæma were drained. He believes that it commenced by an entero colitis leading to pleurisy. This perforated downwards, through the diaphragm, and caused the subphrenic abscess. The signs of pneumothorax and similar signs in the epigastrium were produced by a secondary later perforation of the intestine. Pneumonia in the other lung and phlebitis were also present.

M. Marcel Sée[13] has discussed the subject of diaphragmatic pleurisy, and gives an account of its literature. He lays stress on the supracostal type of breathing, the pyrexia, and the precise locality of the pain as the chief aids to diagnosis. Its causes may be arranged as: (1) Primary—tubercle, injuries, over-indulgence, wounds, and sometimes chill; (2) secondary—general diseases, during which serous surfaces are attacked, such as rheumatism, typhoid, influenza, Bright's diseases; local lesions, from which infection is carried by vascular and lymphatic channels, or spreads by contiguity from any of the thoracic or abdominal organs. Diseases of the female genitals are not an infrequent cause. A chill is taken during menstruation, abdominal pain and fever follow. The pelvic peritonitis may give place to symptoms of diaphragmatic pleurisy, and the streptococcus present be carried by the lymphatics of the uterus up the pillars of the diaphragm, as described by Lasné. Mr. Lawson Tait also has a paper on the subject in the *British Medical Journal* of November.

Primary interlobar pleurisies are uncommon, and not easy to diagnose. Dietrich Gerhardt[14] describes them as accompanied by lancinating pains and dyspnœa; the fever may be high and intermittent. Dulness may be found from the third or fourth dorsal spine over an area outwards and downwards for some distance, with normal resonance above and below it, but physical signs are not well marked in many cases. The expectoration is scanty in the earlier stages, and later on is poured out in large quantities, and smells badly. This increase of expectoration frequently heralds a favourable change. Among the causes of the disease he notices tubercle, pneumonia, and general pleurisy. General septicæmia may develop during its course. Examination by long needles may reveal deeply-seated pus.

Of the value of salicylic acid, its sodium salt and salol in the treatment of pleurisy, there has been much discussion during the last ten years since its recommendation by Aufrecht.[15] A full review of the opinions of German and American authors on the subject was given by Professor G. Dock, together with observations of his own. He remarks that in 32 cases thus treated by Köster, all of more than moderate severity, and some inveterate, aspiration was only needed three times. He himself found evidence of marked diuresis, together with disappearance of the effusion, and on testing the pleural fluid during the treatment demonstrated the presence of salicylates in it. While he acknowledges the rapid spontaneous absorption which sometimes occurs, he thinks that the average duration of treatment is less under salicylates than under any other drugs. They are comparatively harmless, and can be used at an early period in doses of 60 to 90 grains of the soda salt or salol daily, to be reduced to 40 or 30 when their action is evident. Dock himself prefers salol. He adds that they act most promptly in pleurisies with serous effusion, they act efficiently in simple dry pleurisy and often in secondary cases, but there is no evidence that they are of any use in empyæmata.

Casacovice and Sigalea[16] report the favourable result of painting the posterior part of the thorax over an effusion daily with a mixture of guaiacol and tincture of iodine, a drachm and a half of the former to six drachms and a half of the latter for each application. Rapid absorption, with fall of temperature, diuresis, and sweating were observed.

Prince Ludwig Ferdinand[17] has carried on some important researches on the bacteriology of pleurisy to throw light on the question why some exudations disappear and others become purulent. He made cover glass preparations, plate cultures, and inoculations from twenty-three cases. In five cases the diplococcus was found alone, in three the staphylococcus, in five the streptococcus, in two the diplo and streptococcus, in one the staphylo and streptococcus, in two tubercle, and in five no bacteria. In the diplococcus cases the course, even where purulent, was benign, and no cultures grew when taken four days after the crisis of the pneumonia, suggesting the rapid death of the germ. Staphylococcus cases do not always become purulent, but streptococcal ones are very severe, and need early resection. Many cases without bacteria are really tubercular. In diplococcus cases he waits three weeks, and then aspirates first. In staphylococcal serous ones he aspirates, and, if then pus occurs, resects the rib.

Dr. J. Carmichael[18] tabulates the results in forty-eight cases of pleural effusions treated in the Hospital for Sick Children. He believes that pleural effusion is rarely, if ever, primary in nature. The treatment of simple effusions was purely medical, only one case out of twenty-four being aspirated. Out of twenty-four purulent cases five were successfully aspirated, and a free opening was made in nineteen. Simple drainage is alone required in most cases, irrigation or resection of ribs is rarely necessary.

Dr. Fyffe[19] reports a case of non-tubercular double pleurisy in a healthy boy, followed by empyæma on both sides of the chest, and complicated with pneumonia of the left lung, which ended in complete recovery after evacuating the abscesses by operation and drainage. Dr. J. Swain, taking advantage of the amount of plastic exudation which limited the pleural cavities, opened the left side and drained it. Two days later the right cavity was also drained, and after profuse discharge from both sides the sinuses finally closed, and the patient became convalescent in six weeks time.

[1] Med. Chronicle, June, 1893. [2] Lancet, Nov. 25, 1893. [3] Med. Chronicle, Aug., 1893. [4] Br. Med. J., Aug. 19, 1893. [5] Bart's Hosp. Rep., xxviii., p. 133. [6] Lancet, July 22, 1893. [7] Amer. Med. Surg. Bulletin, Sept., 1893. [8] Therapeutic Gazette, Feb. 15, 1893. [9] Br. Med. J., July 19, 1893. [10] Therapeutic Gazette, Dec., 1892. [11] Lancet, July 8, 1893. [12] Rev. de Medic., July, 1893. [13] Quarterly Med. Journal, July, 1893. [14] Berl. Klin. Wochensch., Aug. 14, 1893. [15] Therapeutic Gazette, Feb. 15, 1893. [16] Amer. Med. Surg. Bulletin, Nov., 1893. [17] Amer. J. of Med. Science, I., p. 609, 1893. [18] Edin. Hosp. Rep., I., p. 270, 1893. [19] Bristol Med. Chir. J., p. 233, Dec., 1893.

